# Audiovisual emotion perception develops differently from audiovisual phoneme perception during childhood

**DOI:** 10.1371/journal.pone.0234553

**Published:** 2020-06-18

**Authors:** Hisako W. Yamamoto, Misako Kawahara, Akihiro Tanaka

**Affiliations:** 1 Tokyo Woman’s Christian University, Suginami-ku, Tokyo, Japan; 2 Japan Society for the Promotion of Science, Chiyoda-ku, Tokyo, Japan; University of Hull, UNITED KINGDOM

## Abstract

This study investigated the developmental paths in the use of audiovisual information for the perception of emotions and phonemes by Japanese speakers. Children aged 5 to 12 years and adults aged 30 to 39 years engaged in an emotion perception task in which speakers expressed their emotions through their faces and voices, and a phoneme perception task using phonemic information in speakers’ lip movements and speech sounds. Results indicated that Japanese children’s judgement of emotions by using auditory information increased with increasing age, whereas the use of audiovisual information for judging phonemes remained constant with increasing age. Moreover, adults were affected by visual information more than children. We discuss whether these differences in developmental patterns are due to differential integration processes for information indicative of emotions and phonemes, as well as possible cultural / linguistic reasons for these differences.

## Introduction

Human beings use visual and auditory information to understand others’ intentions during face-to-face communication. It is well known that both emotions and phonemes are perceived through multisensory inputs. The face and the voice convey the speakers’ emotions and integrating these cues enables us to recognize the emotions of others [1]. Recent research has demonstrated cultural differences in integrating audiovisual information for the perception of emotions. For example, Tanaka, et al. [2] demonstrated that perception of emotions by Japanese people tends to be influenced by vocal expressions, whereas Dutch people put more weight on facial expressions, suggesting that Japanese people use auditory cues for the perception of emotions more than Dutch people. However, Kawahara, et al. [3] suggested that 5-6-year-old Japanese children tend to judge emotions by the speakers’ facial expressions more than by their vocal expressions. Therefore, age differences might be observed in the use of auditory and visual information for the perception of emotions by Japanese people.

Phonemes are perceived through lip movements as well as speech sounds. The McGurk effect [4] is a well-known phenomenon in which the sound /da/ is perceived when the voice /ba/ is played over the facial movements for /ga/. Just as for impact of culture on audiovisual emotion perception, the influence of visual information for the perception of phonemes differ depending on the listeners’ linguistic background. For example, the McGurk effect is less frequently induced in Japanese speakers than in English speakers [5], and this difference increases gradually during childhood [6]. These studies suggest that Japanese speakers tend to be less influenced by lip movements for phoneme perception than English speakers, suggesting that Japanese speakers might put greater weight on auditory information when perceiving phonemes.

It has been suggested that Japanese people tend to be less influenced by visual information and put more weight on auditory information than Western people in the perception of emotions and phonemes [2–5]. This difference has been explained from the viewpoint of cultural [2][3] and linguistic factors [5][6], suggesting that such a style of audiovisual perception is acquired through development. However, it remains unclear whether the developmental patterns of using audiovisual information to perceive emotions and phonemes are similar or different. Therefore, we investigated the developmental paths of Japanese people’s use of audiovisual information to perceive emotions and phonemes to reveal how they acquire their cultural/language-specific processes in audiovisual perception.

We focus in the present study on the development between 5 to 12 years old. Previous developmental studies have suggested that children’s perception style develops during their school-age years. Sekiyama and Burnham’s study [6] showed that the occurrence of the McGurk effect increased between 6 to 11 years old in English-speaking children. Also, regarding emotion perception, one study revealed age differences in the perception of emotional prosody in the voice between 5–7 years and 8–10 years [7]. Considering the importance of development during school age, we compared audiovisual perception styles between Japanese children aged 5–12 years and adults.

## Materials and methods

### Ethics statement

The protocol of this study is approved by Tokyo Woman’s Christian University Research Ethics Committee. All the adult participants and the child participants’ parents or guardians were informed about the purpose of the study and gave written informed consent in accordance with the Declaration of Helsinki.

### Participants

The participants were 5-6-year-old (*n* = 82 [34 girls], *Mage* = 5.6 years; *SD* = 0.5), 7-8-year-old (*n* = 92 [50 girls], *Mage* = 7.5 years; *SD* = 0.5), 9-10-year-old (*n* = 95 [36 girls], *Mage* = 9.5 years; *SD* = 0.5), and 11-12-year-old (*n* = 80 [35 girls], *Mage* = 11.4 years; *SD* = 0.5) children, as well as 30 to 39-year-old adults (*n* = 45 [18 women], *Mage* = 34.7 years; *SD* = 2.8). None of adult participants had raised their own children. All participants were native Japanese speakers recruited at the National Museum of Emerging Science and Innovation (Miraikan) in Tokyo, Japan.

### Apparatus

The experimenters used a 15-inch laptop computer to present and control audiovisual stimuli by using Hot Soup Processor software version 3.4 (Onion software). Participants were seated in front of the laptop computer at a distance of approximately 50 cm. The visual stimuli were displayed in the middle of the monitor at a 640×480-pixel size (the actual size of the display was 16.3×12.2 cm). Sound stimuli were presented through headphones (HDA300, SENNHEISER) at a maximum volume of approximately 70 dB SPL, which was adjusted to mask the background noise in the laboratory by using amplifiers in the headphone (DAC-HA200, ONKYO).

### Stimuli and procedure

Participants conducted emotion perception and phoneme perception tasks in a laboratory of Miraikan. In both tasks, they were presented with audiovisually congruent and incongruent stimuli as follows.

#### Emotion perception task

**Fig 1:**
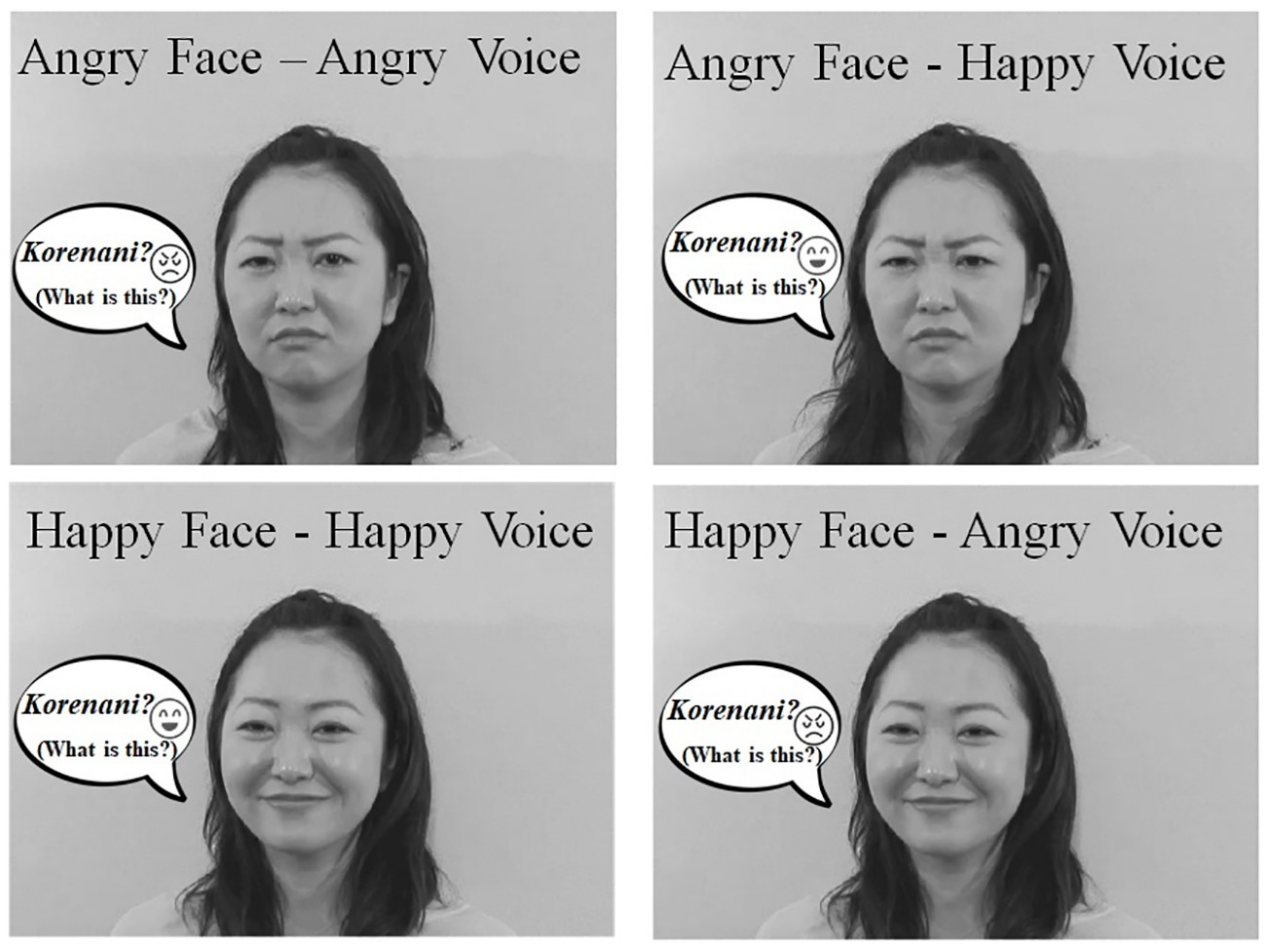
Sample images of stimuli in the emotion perception task.

The audiovisual stimuli of the emotion perception task were chosen from stimuli of Tanaka et al ‘s study [1] with a modification. Facial images were degraded with dynamic noises to adjust the difficulty of tasks in their previous study, while we used original recorded videos without visual noises in the present study. Moreover, we picked only Japanese speakers’ stimuli. Consequently, the stimuli used in present study consisted of short movies in which 21-year-old female Japanese speakers expressed happiness or anger in their face and voice (Fig 1). The linguistic information in the voices was emotionally neutral and included statements such as “*Hai*, *moshimoshi*” (Hello), “*Sayonara*” (Good-bye), “*Korenani*” (What is this?), and “*Sounandesuka*” (Is that so?). Congruent stimuli were original recorded video clips, while to create the incongruent stimuli, angry (happy) voice sounds were dubbed onto happy (angry) face video clips. A total of 32 movies (two speakers × four emotions (angry face and voice, happy face and voice (congruent), angry face and happy voice, happy face and angry voice (incongruent)) × four utterances) were used as test stimuli. The average duration of the test stimuli was 2017 ms (range: 1602–2436 ms). In addition to them, two other movies were used as practice stimuli. In each trial, a fixation point was displayed at the center of the monitor for 500 ms, simultaneously with an auditory signal (440 Hz pure tone lasting 100 ms). After 500ms from the onset of the fixation, the movie, and a blank display were presented successively. Participants were asked to judge whether the woman was happy or angry and respond by pressing the D or K keys of the keyboard. The next test trial began 500 ms after the participant’s response. Following two practice trials, a total of 32 test trials that included 16 congruent and 16 incongruent trials was conducted. The order of test trials was randomized.

#### Phoneme perception task

The phoneme perception task was conducted after the emotion perception task. To minimize the variance of the data within each task, we kept the task order constant.

**Fig 2:**
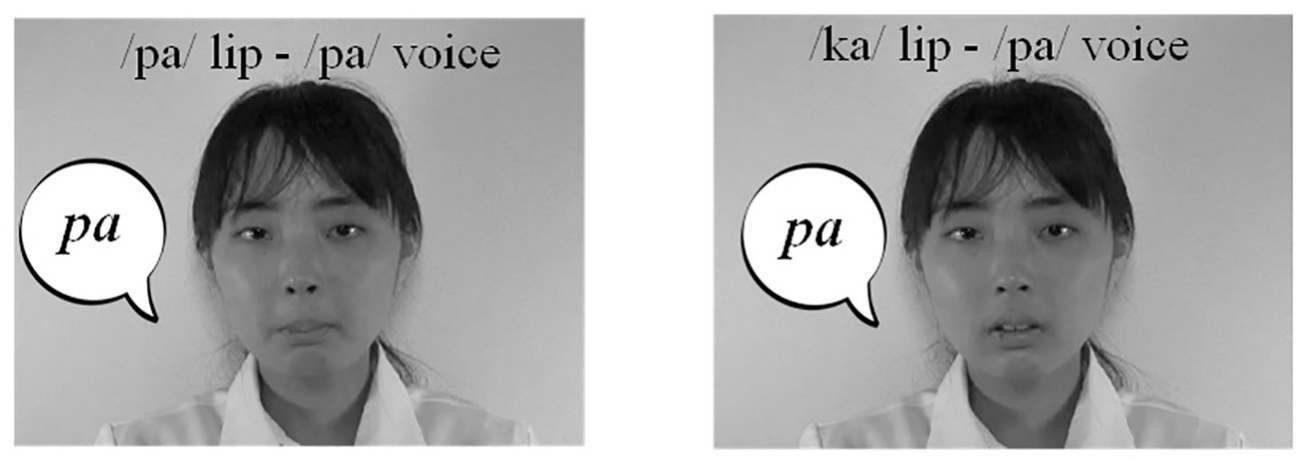
Sample images of stimuli in the phoneme perception task.

The audiovisual stimuli of the phoneme perception task were previously used in Tanaka, Asakawa and Imai’s study [8]. These audiovisual stimuli were short movies in which a male or female Japanese speaker (Fig 2) pronounced one syllable (/ka/, /pa/, or /ta/). All speakers were in their twenties (*M* = 24.7 years old, range: 22–28 years old) when the video clips were recorded, and they did not participate in the recording of the stimuli of the emotion perception task. Test stimuli contained twelve congruent movies (lip movement and sound were congruent) and six McGurk type incongruent movies (/ka/ lip movement combined with /pa/ sound). As with the emotion perception task, congruent stimuli were original recorded video clips, while in the incongruent stimuli, /pa/ voice sounds were dubbed onto /ka/ mouth video clips. The average duration of the stimuli was 996 ms (range: 834–1235 ms).

In our preliminary experiment with adults, only small visual influences were observed when the fixation point was presented for 500 ms as in the emotion perception task, suggesting that this duration was insufficient for the phoneme perception task. To avoid a floor effect on visual influences, we set the duration of the presentation of a fixation point and the blank in the phoneme perception task longer so that the children could move their eye gaze around the fixation before the onset of visual stimuli and examine the audiovisual phoneme perception at a more appropriate length.

A fixation point was displayed at the center of the monitor for 800 ms in each trial, simultaneously with an auditory signal (440 Hz pure tone lasting 100 ms), then a blank display was presented for 500 ms. After 1300ms from the onset of the fixation, the movie, and a blank display were presented successively. Participants were asked to judge whether the speaker said /ka/, /pa/, or /ta/ and respond by pressing Z, V or M keys. The next test trial began after 500 ms from the participant’s response. Each congruent movie was presented once (12 congruent trials), whereas each McGurk type movie was presented twice (12 McGurk trials), resulting in a total of 24 trials. The order of test trials was randomized.

## Results

### Emotion perception task

**Fig 3:**
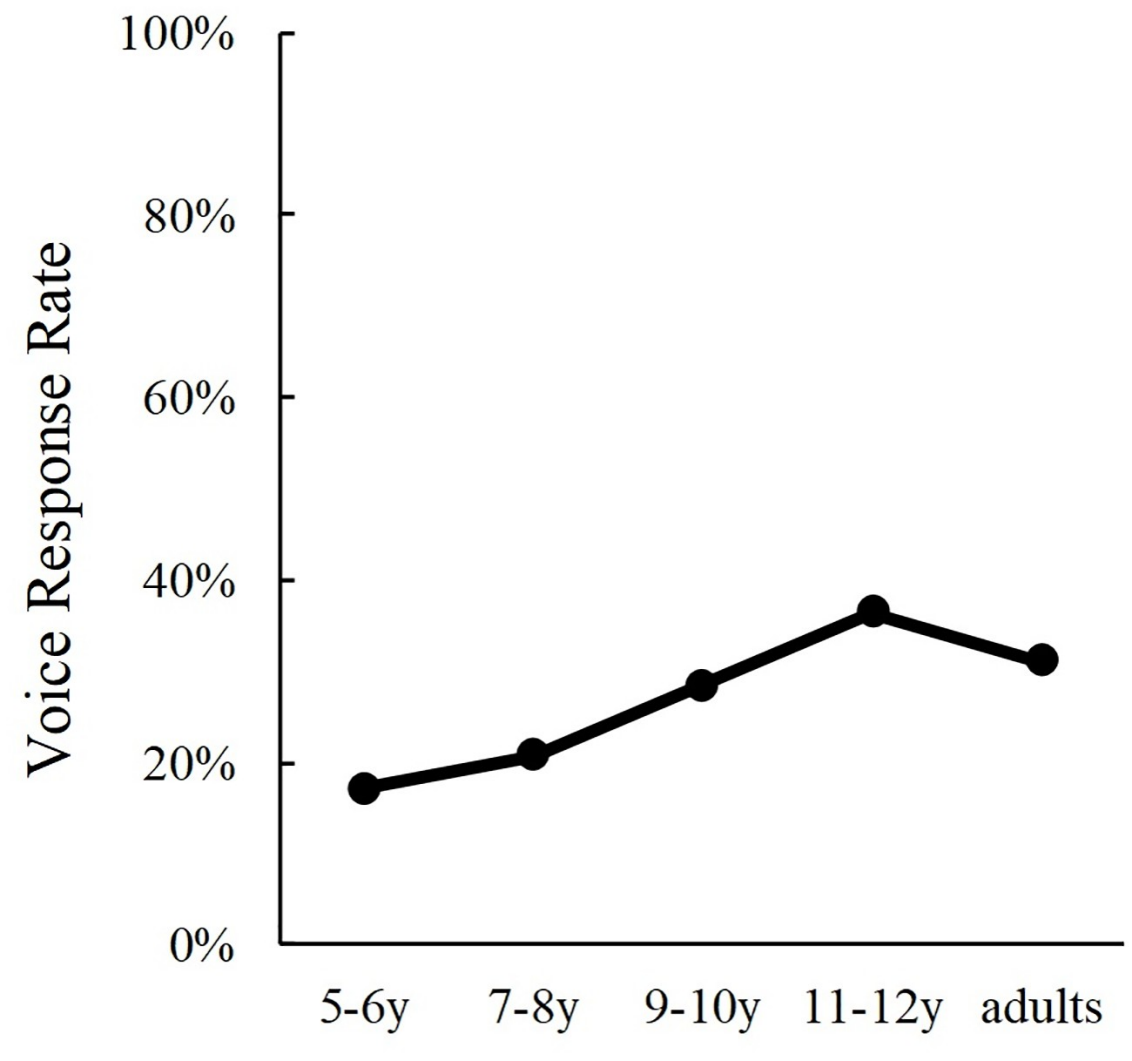
Developmental changes in auditory response rates in the emotion perception task.

The auditory responses, which indicated the participants’ rate of making judgements of emotions based on the speaker’s voice in incongruent trials is shown in Fig 3. We conducted a one-way analysis of variance (ANOVA) of auditory response rates to examine for age differences. Results indicated a significant main effect of Age (*F*(4, 389) = 11.57, *p* < .001, *η*_*p*_^*2*^ = .11). The post hoc analysis (Shaffer’s modified sequentially rejective Bonferroni procedure) revealed that 5- to 6-year-olds made significantly less auditory responses in incongruent trials than 9- to 10-year-olds (*p* = .002), 11- to 12-year-olds (*p* < .001), or adults (*p* = .002). Furthermore, 7- to 8-year-olds made significantly less auditory responses than 9- to 10- year-olds (*p* = .048), 11- to 12- year-olds (*p* < .001), or adults (*p* = .035). Furthermore, the auditory responses of 9- to 10-year-olds were less than those of 11- to 12- year-olds (*p* = .037). The results of incongruent trials suggested that Japanese children come to judge emotions by putting more weight on information from the voice with increasing age when the emotions expressed by the face and the voice are different.

**Fig 4:**
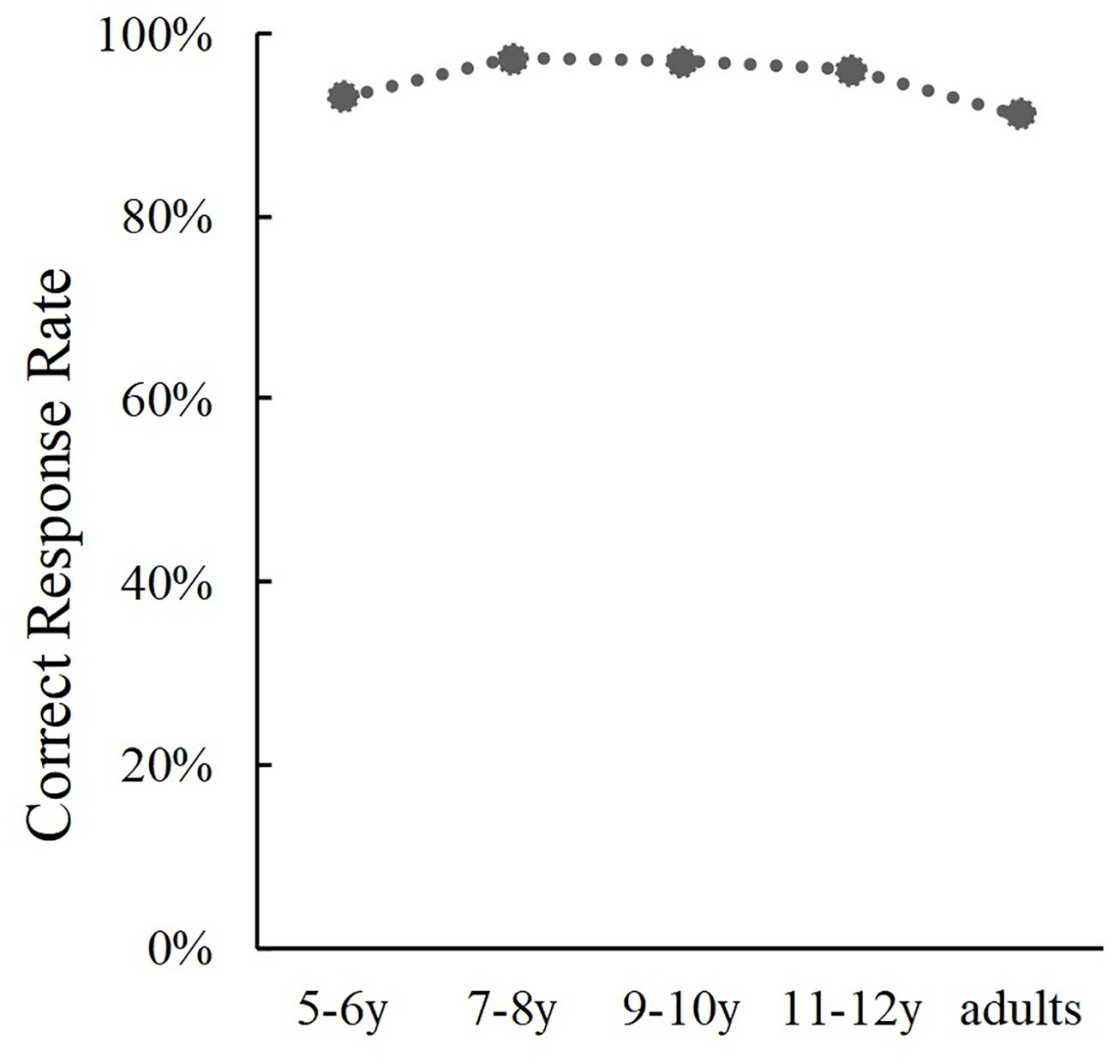
Developmental changes in correct response rates in the emotion perception task.

As for congruent trials, we conducted a one-way ANOVA of the correct response rates (Fig 4). Results indicated a significant main effect of Age (*F*(4, 389) = 5.44, *p* < .001, *η*_*p*_^*2*^ = .05). The post hoc analysis revealed that 5- to 6-year-olds made significantly less correct response rate than 7- to 8- year-olds (*p* = .017) and 9- to 10-year-olds (*p* = .032). Moreover, adults made significantly less auditory responses than 7- to 8- year-olds (*p* = .003), 9- to 10-year-olds (*p* = .003), or 11- to 12- year-olds (*p* = .028). These results indicated that the accuracy of emotion judgements from the face and the voice increased during childhood, but slightly decreased after adolescence. The performance under each kind of stimulus is shown in Table 1.

**Table 1:** Average performances in the emotion perception task (%).

	Congruent (Correct Response Rate)				Incongruent (Voice Response Rate)			
	Angry Face/Angry Voice		Happy Face/Happy Voice		Angry Face/Happy Voice		Happy Face/Angry Voice	
5-6y	92.5	(13.4)	93.6	(14.2)	15.2	(22.7)	19.1	(19.8)
7-8y	98.0	(5.3)	96.6	(6.7)	18.1	(24.5)	23.5	(21.4)
9-10y	98.9	(3.5)	95.0	(9.2)	18.8	(22.8)	37.9	(28.4)
11-12y	98.9	(4.1)	93.1	(15.0)	24.2	(28.0)	48.8	(29.3)
adults	95.0	(16.5)	87.2	(23.0)	12.8	(21.9)	49.4	(29.7)

Standard deviations are given in parentheses.

For further analysis, we conducted an Age × Emotion (angry face and happy voice, happy face and angry voice) mixed-factor analysis of variance (ANOVA) of auditory response rates in incongruent trials to examine the effect of the combination of facial and vocal emotions. Results indicated a significant interaction between Age and Emotion (*F*(4, 389) = 14.91, *p* < .001, *η*_p_^2^ = .13). A simple main effect analysis showed that auditory responses were significantly different between age groups for happy face and angry voice (*F*(4, 389) = 22.04, *p* < .001). A post hoc analysis (*p*s < .001) revealed that auditory responses were less frequently observed in the 5- to 6- year-olds, 7- to 8- year-olds, and 9- to 10- year-olds groups than 11- to 12- year-olds and adults. Auditory responses were also less frequent in the 5- to 6- year-olds and 7- to 8- year-olds groups than 9-to 10- year-olds. The simple main effect of Age was only marginally significant for auditory response rates for angry face and happy voice (*F*(4, 389) = 2.12, *p* = .077). This additional analysis suggests that Japanese children come to judge emotion as angry when people speak with an angry voice even though they express happiness in their faces (Table 1).

### Phoneme perception task

**Fig 5:**
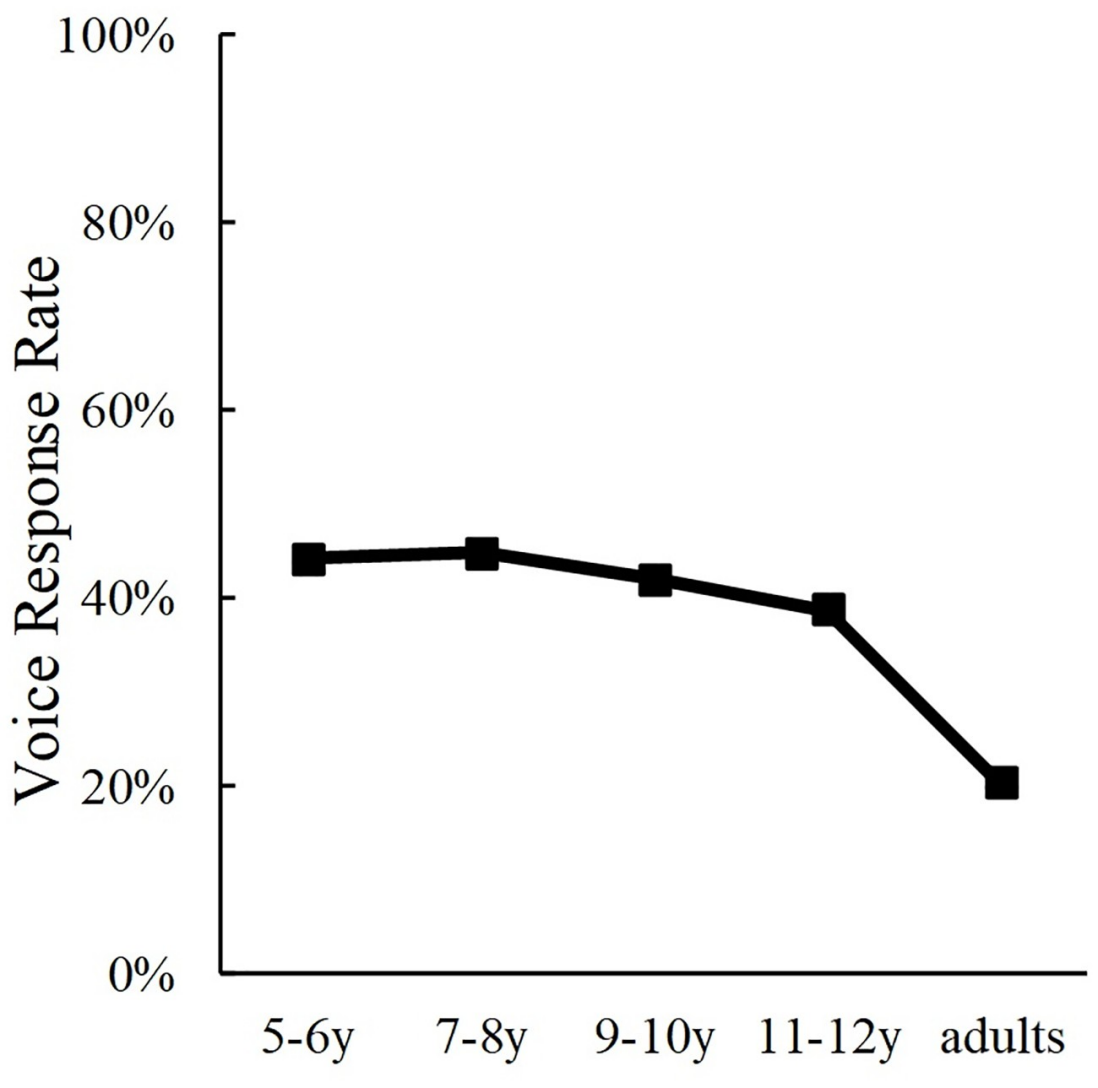
Developmental changes in auditory response rates in the phoneme perception task.

We regarded participants’ /ka/, /ta/, /pa/ responses in incongruent trials respectively as visual, fusion, and auditory responses. The auditory responses that indicate the rates of participants’ phoneme judgement based on the speaker’s voice are shown in Fig 5. We conducted a one-way ANOVA of auditory response rates to examine for age differences. Results indicated a significant main effect of Age (*F*(4, 389) = 9.11, *p* < .001, *η*_*p*_^*2*^ = .09). Auditory responses of adults were less than the other groups (*ps* < .001) in incongruent trials, whereas differences in auditory responses among the children-groups were non-significant. These results suggested that the use of visual information about lip movements remain constant during childhood, whereas it increases after adolescence.

Moreover, we also conducted a one-way ANOVA of fusion response rates (Table 2), and found a significant main effect of Age (*F*(4, 389) = 15.81, *p* < .001, *η*_p_^2^ = .14). Adults had more frequent fusion response rates (*ps* < .001) than the other groups, and no significant differences were observed among the children’s groups.

**Table 2:** Average performances in the phoneme perception task (%).

	Congruent (Correct Response Rate)						Incongruent					
	Ka		Ta		Pa		*Auditory Response Rate*		*Fusion Response Rate*		*Visual Response Rate*	
5-6y	90.9	(17.8)	94.8	(13.5)	93.3	(13.6)	44.1	(25.2)	46.0	(24.5)	9.9	(11.8)
7-8y	95.1	(11.3)	94.6	(11.6)	98.6	(6.8)	44.7	(25.2)	46.2	(24)	9.1	(10.8)
9-10y	95.5	(10.3)	95.8	(12.4)	96.3	(12.6)	41.9	(24.6)	48.5	(22.6)	9.6	(9.5)
11-12y	96.9	(9.2)	96.9	(10.8)	98.1	(9.6)	38.6	(22.7)	52.4	(21.3)	9.0	(10.2)
adults	96.1	(10.6)	99.4	(3.7)	100.0	(0)	20.2	(23.3)	76.5	(23.5)	3.3	(6.3)

Standard deviations are given in parentheses.

**Fig 6:**
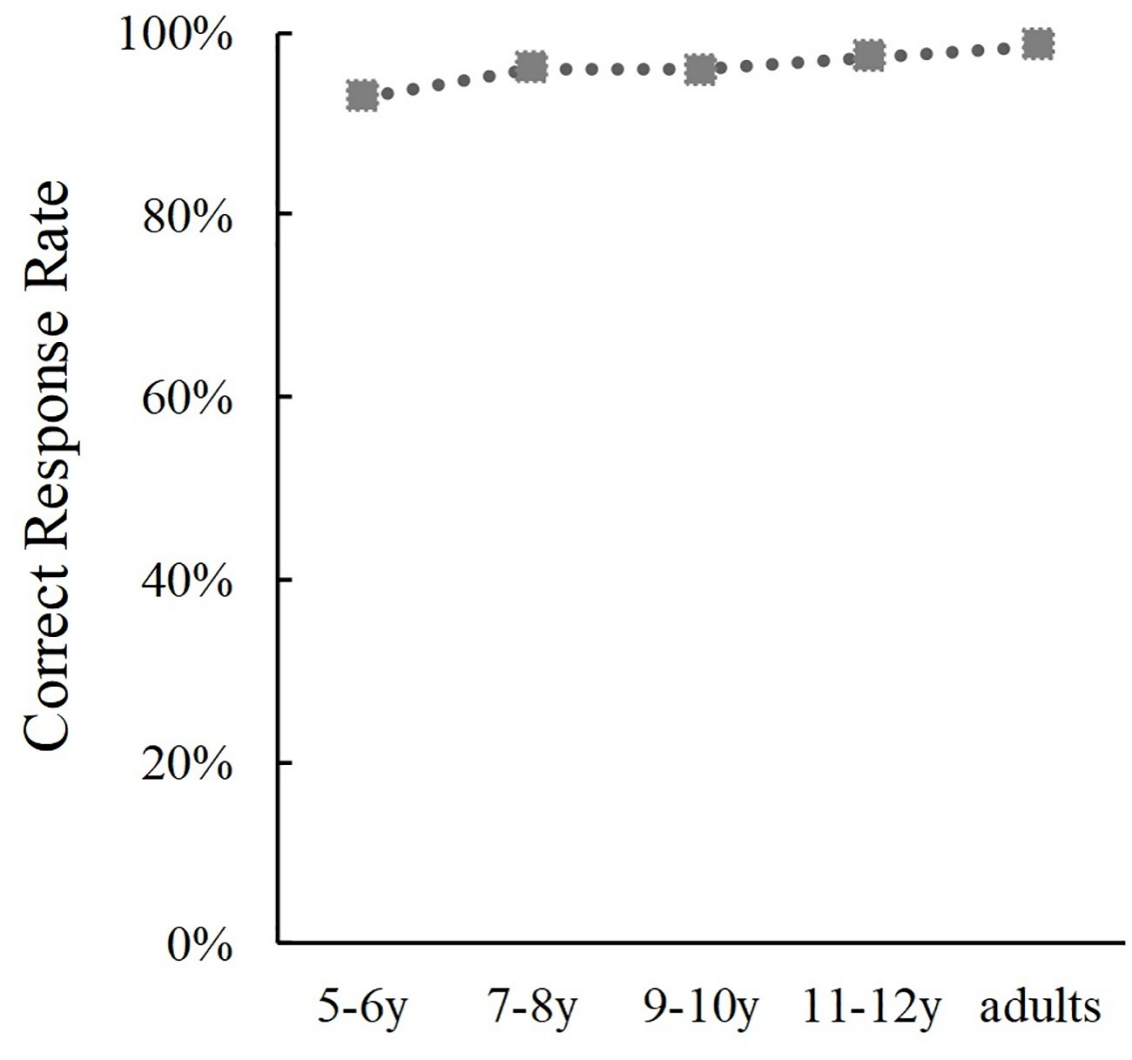
Developmental changes in correct response rates in the phoneme perception task.

For congruent trials, we conducted a one-way ANOVA of the correct response rates (Fig 6). Results indicated a significant main effect of Age (*F*(4, 389) = 5.03, *p* < .001, *η*_p_^2^ = .50). The post hoc analysis revealed that correct responses of 5-6-year-olds were less than those of 7-8-year-olds (*p* = .043), 11-12-year-olds (*p* = .002) or adults (*p* = .001). Additionally, the difference between correct response rates of 5-6-year-olds and 9-10-year-olds were marginally significant (*p* = .073), which indicated that phoneme judgement accuracy from the face and voice increased slightly from the of age 5–6 years when the lip movement and sound were congruent.

Finally, we calculated Pearson’s correlation coefficients between voice response rates in incongruent trials for emotion and phoneme perception tasks to explore relationships between the perception of emotions and phonemes using audiovisual information. Results indicated that the partial correlation was not significant when we controlled for each child’s age (*r* = .03). Moreover, no significant correlations were found between voice response rates in incongruent trials for the two tasks in each group (Table 3). Thus, the tendency of getting weight to auditory information in the audiovisual perception of emotions is not correlated with that of phonemes.

**Table 3:** Correlation coefficients between voice response rates in incongruent trials for the two tasks in each age group.

5-6y	.08
7-8y	.01
9-10y	.06
11-12y	.13
adults	.02

## Discussion

The results of this study indicated that Japanese people’s perception of emotions and phonemes by using audiovisual information develops along different paths. The relative influence of vocal expressions increased and the relative influence of facial expressions decreased during childhood in the case of emotions. When perceiving the emotions of others, young Japanese children tend to focus only on the speaker’s facial expressions. However, they come to judge others’ emotions by placing greater weight on vocal expressions, not only on facial expressions with increasing age.

As shown in previous studies [9–11], facial expressions are perceived more accurately than vocal expressions. The results of the present study also showed that facial cue is still dominant even in older children and adults. Nevertheless, the relative reliance on facial cue tends to decrease with perceivers’ age among Japanese people. This may be caused by their emotion expression style. Japanese people tend to express their emotions with their face, less often than Western people, and this difference is more pronounced for negative emotions [12]. For this reason, the voice could be a better source of useful information for Japanese people for precisely recognizing the hidden emotions of others. This may be salient when the speakers intend to hide their negative emotion with smiles, given the difference of auditory response rates between happy face—angry voice and angry face—happy voice. Japanese children may learn this culture-specific display pattern and the decoding rule [13][14] through daily communication with members of their community including friends, family, and teachers, and come to interpret others’ emotions by taking vocal emotions in addition to facial expressions into consideration.

In contrast to such developmental change in the perception of emotions, we did not observe a developmental change in the perception of phonemes during childhood. This finding is consistent with Sekiyama and Burnhams’ study demonstration that the influence of vision increased with increasing age in English-speaking children, whereas such a developmental change was not observed in Japanese-speaking children [6]. Our results also demonstrated that Japanese adults were more affected by lip movements than Japanese children. These results suggest that Japanese children come to integrate audiovisual information later in life for the perception of phonemes more than for the perception of emotions.

To summarize, this study demonstrated that the developmental path of using audiovisual information differs for the perception of emotions and the perception of phonemes in two aspects. First, they occur at different times of development. Developmental changes in the perception of emotions using audiovisual information occur during childhood, whereas the perception of phoneme does not change until later in life. Second, the direction of these developmental changes is different. The influence of visual information in the perception of emotions decreases with increasing age, whereas this direction is opposite for the development of phoneme perception, in which the influence of visual information increases after childhood.

What is the cause of these developmental differences? Several factors might cause the differences between the perceptions of emotions and phonemes. The first is the effect of the manner of communication. The ways of expressing emotions are culturally diverse [12]. Therefore, the judgment of emotions depends on the display and decoding rules of the perceivers’ environment. As a result, developmental changes in using audiovisual information for the perception of emotions compared to the perception of phonemes might be more strongly related to acquiring manners of communication. This difference could result in children changing cues they use for the perception of emotions. The manner of communication seems to be irrelevant for the perception of phonemes. This could be because people have to infer others’ emotions by considering that others might hide their real emotions in social situations, whereas this inference is unnecessary for the perception of phonemes. On the other hand, the perception of phonemes using audiovisual information might be more affected by linguistic characteristics, such as the number of phonetic inventory, rather than by the cultural context.

Second, the development of unimodal perception might differ between the perception of emotions and the perception of phonemes. The visual skills needed for lipreading when perceiving phonemes and the visual skills needed to distinguish between facial expressions are different [15]. A similar difference can be seen in auditory perception. Phoneme and other linguistic information are conveyed through segmental features, whereas emotions and other paralinguistic information are mostly conveyed through the suprasegmental features of vocal expressions. It would be worthwhile to investigate the relationship of unimodal perception skills with the perception of emotions and phonemes in Japanese speakers by using audiovisual information in future studies.

Third, we should consider the possibility that cultural and linguistic factors affect the development of audiovisual perception differently. For example, cultural factors may modulate the relative importance of auditory / visual information (in the case of Japanese, culture increases the importance might be tested in subjects whose cultural background is fully Japanese but of auditory information), while linguistic factors may boost the utilization of visual information. To examine this possibility, we need to dissociate the cultural and linguistic factors. This who do not speak Japanese, or with people in other East Asian countries.

There are some limitations in the present study. First, the results obtained here are limited to subjects aged 5–12 and 30–39 years old. It should be noted that adult participants in the present study (30–39 years) were older than those in Sekiyama and Burnham’s [6] previous study (18–29 years). In another study examining the impact of aging on audiovisual phoneme perception, 60–65-years-olds were more influenced by visual information than 19–21-year-olds [16]. According to such an aging effect, audiovisual perception style may change throughout adulthood as well as childhood. Considering age-related impairments in the perception of affective prosody [17][18], it is possible that the weighting of auditory information in audiovisual emotion perception changes in an inverted V-shape across the life span. To explore such lifelong development, it is worth investigating audiovisual perception in various generations.

Second, as mentioned in the Methods section, we adopted the constant task order to minimize the variance within each task. Thus, we cannot rule out the possibility that the emotion perception task affects performance on the phoneme perception task (e.g., the preceding task might induce attention to visual or auditory modality, or better performance in the latter task). We chose the constant task order because our primary aim is to compare differences across age groups. Further study is needed directly comparing the results of the two tasks regarding the balance of modalities (e.g., comparing the auditory response rate in the emotion perception task with that in the phoneme perception task) by counterbalancing the task order.

Third, we should note that speakers’ gender was different between the emotion and phoneme tasks. Considering that there are gender differences in facial expressions [19] and that the fundamental frequency of the voice differs between the genders, the speakers’ gender may affect audiovisual perception. Further study is needed examining this gender effect.

This study demonstrated different developmental paths in the perception of emotions and phonemes by using audiovisual information, which suggested different processes of integrating such information. It is, however, possible that the perceivers’ communication habits such as fixating on the other’s face affected the perception of emotions and phonemes using audiovisual information, even though the processes were different, which is a possibility that needs to be investigated in future studies. It would also be useful to investigate cultural differences in the expression of emotions using audiovisual information in naturalistic situations and explore how cultural differences in the perception of emotions reflect differences in the expression of emotions.

## Supporting information

S1 Data (XLSX)
